# High Cardio-Ankle Vascular Index Values in Idiopathic Sudden Sensorineural Hearing Loss Patients Indicate Better Prognosis

**DOI:** 10.7759/cureus.49400

**Published:** 2023-11-25

**Authors:** Munetaka Ushio, Yoshihisa Kitazawa, Yuya Tamura, Tomoe Yoshida, Michiko Uchiyama, Taro Takanami, Toshitake Tanaka, Yoshihiro Ikemiyagi, Fuyuko Ikemiyagi, Yasushi Ohta

**Affiliations:** 1 Otolaryngology, Toho University Sakura Medical Center, Sakura, JPN; 2 Otolaryngology, Kitazawa Ear Nose Throat Clinic, Tokyo, JPN; 3 Otolaryngology, Matsudo Ear Nose Throat Clinic, Matsudo, JPN; 4 Clinical Support Service, Toho University Sakura Medical Center, Sakura, JPN; 5 Otolaryngology, Tokyo Metropolitan Police Hospital, Tokyo, JPN; 6 Otolaryngology, Ikemi Ear Nose Throat Clinic, Narita, JPN

**Keywords:** vascular disorders, prognosis, idiopathic sudden sensorineural hearing loss, cavi, cardio-ankle vascular index

## Abstract

Objective: Vascular disorders and viral infections are the presumed etiologies of idiopathic sudden sensorineural hearing loss (ISSNHL) and acute sensorineural hearing loss, with no identifiable cause. However, no clinical test for estimating the extent of vascular involvement in ISSNHL has been reported despite its potential impact on prognosis and treatment. We investigated the correlation between the cardio-ankle vascular index (CAVI), which reflects arterial stiffness and elasticity, and hearing improvement to ascertain its usefulness as an additional indicator of ISSNHL prognosis and etiology.

Methods: We enrolled 182 patients diagnosed with definite ISSNHL. The percentage of mild ISSNHL patients and that of patients experiencing no change were compared between the high-CAVI and low-CAVI groups. Age, initial and final pure-tone average (PTA) values, CAVI, presence or absence of vertigo, and medical histories were retrospectively reviewed and included in univariate and multivariate analyses.

Results: The percentage of mild ISSNHL patients was smaller in the high-CAVI group than in the low-CAVI group, whereas the percentage of patients experiencing no change was smaller in the high-CAVI group than in the low-CAVI group, although patients in the high-CAVI group were significantly older than those in the low-CAVI group. The Cox proportional hazard model revealed that high CAVI, hypertension, younger age, and initial PTA <90 dB were associated with hearing improvement.

Conclusions: ISSNHL in patients with high CAVI was more severe but had a better prognosis than that in those with low CAVI. CAVI may help evaluate diseases of vascular and other etiologies, as well as ISSNHL.

## Introduction

For diseases with multiple possible etiologies, such as vascular disorders, viral infection (e.g., vestibular neuritis), and diabetes mellitus- and age-related cerebral nerve palsy, effective treatment requires accurate etiological identification at clinics for effective prognostication and management. Idiopathic sudden sensorineural hearing loss (ISSNHL) is an acute disease resulting from abnormal function of the cochlea, auditory nerve, or higher aspects of the central auditory system, with no identifiable cause [1,2]. ISSNHL affects approximately 5-61 individuals per 100,000 people annually in the United States [3], Taiwan [4], Switzerland [5], and Japan [6]. The etiology of ISSNHL remains unclear [7], although probable causes include arteriosclerosis, vasoconstriction [8,9], and viral infections, such as those caused by mumps virus [10], varicella-zoster virus [11], and enterovirus [12]. Viral tests, such as herpes simplex virus types 1 and 2 and varicella-zoster virus antibody tests, have been used to assess viral infection in ISSNHL [12,13]. However, no clinical tests for assessing vascular disorders in this context have been reported.

Arteriosclerosis contributes to various vascular disorders, including cardiovascular and cerebrovascular diseases [14]. Arterial stiffness is a property that accompanies the progression of arteriosclerosis [15] and indicates the extent of vascular disorders. Pulse wave velocity (PWV) is used to evaluate arterial stiffness. The cardio-ankle vascular index (CAVI) developed in 2006 in Japan is a non-invasive index that reflects arterial stiffness [16]. Unlike PWV, CAVI is less susceptible to cardiac function and transient blood pressure changes [17], and is positively correlated with the number of microvascular lesions in the brain [18,19], such as silent brain infarction and white matter hyperintensities on magnetic resonance imaging and the severity of coronary atherosclerosis [20]. The higher the CAVI value, the higher the incidence of cardiovascular events [21]. Thus, CAVI can be employed to evaluate diseases caused by arterial stiffness.

To the best of our knowledge, this report is the first to assess arterial stiffness and elasticity to predict the prognosis and etiology of ISSNHL. This study aimed to investigate the correlation between CAVI and hearing improvement to ascertain its usefulness as an additional indicator of ISSNHL prognosis and its etiology.

## Materials and methods

This retrospective, non-randomized, single-group study conducted at the Toho University Sakura Medical Center was approved by the institutional ethics committee (approval number S21026). Informed consent was obtained from all the patients. The project conformed to the Code of Ethics of the World Medical Association (Declaration of Helsinki).

Participants

From January 2013 to February 2021, 1320 consecutive patients (mean age, 57.3±17.7 years; 679 women and 641 men) with acute sensorineural hearing loss were referred to our clinic in Sakura, Chiba.

The diagnostic criteria of ISSNHL according to the American Academy of Otolaryngology-Head and Neck Surgery Foundation’s guidelines [2] and the Guidelines of the Research Committee of the Ministry of Health, Labor and Welfare of Japan [6] were adopted. The main symptom was the sudden onset of sensorineural hearing loss of ≥30 dB over three consecutive frequencies that occurred within a 72-h window, with no identifiable cause despite adequate investigation. Hearing loss is usually unilateral but may be bilateral at the onset. Hearing loss may be accompanied by vertigo, nausea, and/or vomiting, without recurrent episodes. No cranial nerve symptoms were observed other than those related to cranial nerve VIII.

Patients were excluded if they visited on or after day 15 from disease onset; had been treated with steroids at a previous clinic; were diagnosed with cerebellopontine angle tumor on brain magnetic resonance imaging; underwent insufficient examination, including CAVI assessment; or had acute low-tone sensorineural hearing loss because they had a better prognosis than patients with ISSNHL, representing other hearing loss types [22,23]. Patients with acute low-tone sensorineural hearing loss were diagnosed when the sum of hearing levels at 125, 250, and 500 Hz was ≥70 dB and that of hearing levels at 2000, 4000, and 8000 Hz was ≤60 dB [24].

Data collection

Baseline parameters included age, sex, height, weight, body mass index assessed by the clinical staff, systolic (sBP) and diastolic (dBP) blood pressure, heart rate, CAVI, diabetes mellitus rate, hypertension rate, dyslipidemia rate, and history of diabetes mellitus, hypertension, or dyslipidemia. Diabetes mellitus was defined as fasting plasma glucose levels of ≥126 mg/dL and/or 75-g oral glucose tolerance test with 2-h plasma glucose levels of ≥200 mg/dL and/or random plasma glucose levels of ≥200 mg/dL. Hypertension was defined as sBP ≥140 mmHg and/or dBP ≥90 mmHg. Dyslipidemia was defined as total cholesterol levels of ≥220 mg/dL, high-density lipoprotein cholesterol levels of <40 mg/dL, low-density lipoprotein cholesterol levels of ≥140 mg/dL, and/or triglyceride levels of ≥150 mg/dL. Patients treated with antidiabetic, antihypertensive, or lipid-lowering agents were diagnosed and included.

Measurement and evaluation of the hearing level

All patients underwent otoscopic examination, including management of excessive or obstructive cerumen. At the initial examination, air and bone conduction thresholds were measured with appropriate masking using an audiometer (Audiometer AA-78, RION Co. Ltd, Tokyo, Japan) once a week until two months after disease onset. Subsequent measurements were obtained once every three to four weeks until six months after the onset and once every two to three months thereafter, until the hearing stabilized or complete hearing recovery or stabilization, defined as the difference between two consecutive pure-tone averages (PTA) within 10 dB, was observed. PTA is the arithmetic mean of five frequencies (250, 500, 1000, 2000, and 4000 Hz) on pure-tone audiometry to evaluate the grading of hearing loss and level of hearing recovery [6]. Grading of hearing loss in ISSNHL was defined as follows: Grade 1, PTA < 40 dB; Grade 2, 40 dB ≤ PTA < 60 dB; Grade 3, 60 dB ≤ PTA < 90 dB; and Grade 4, 90 dB ≤ PTA (Table 1) [6].

**Table 1:** Criteria for the grading of hearing loss in ISSNHL [6] PTA: pure tone average (arithmetic mean of the five frequencies of 250, 500, 1000, 2000, and 4000 Hz); dB: decibel; ISSNHL: idiopathic sudden sensorineural hearing loss

Grade	Criteria
1	PTA < 40 dB
2	40 dB ≤ PTA < 60 dB
3	60 dB ≤ PTA < 90 dB
4	90 dB ≤ PTA

The level of hearing recovery was defined as follows: complete recovery, all five frequencies in the final audiograms were ≤20 dB or improvement to the same degree of hearing on the unaffected side; marked improvement, PTA improvement ≥30 dB; slight improvement, 10 dB ≤ PTA improvement <30 dB; and no change, 10-dB PTA improvement (Table 2) [6].

**Table 2:** Hearing improvement criteria for ISSNHL [6] PTA: pure tone average (arithmetic mean of the five frequencies of 250, 500, 1000, 2000, and 4000 Hz); dB: decibel; ISSNHL: idiopathic sudden sensorineural hearing loss

Hearing improvement status	Criteria
Complete recovery	All five frequencies in the final audiograms are 20 dB or less, or improvement to the same degree of hearing in the unaffected side
Marked improvement	PTA improvement ≥ 30 dB
Slight improvement	10 dB ≤ PTA improvement < 30 dB
No change	10 dB < PTA improvement

Measurement and evaluation of CAVI

CAVI was measured once with the VaSera CAVI instrument (Fukuda Denshi Inc., Tokyo, Japan) [16]. Briefly, cuffs were applied to the bilateral upper arms and ankles with the patients in the supine position and the head held in the midline position. After resting for 10 min, 30-50 mmHg low cuff pressure was used to detect the brachial and ankle pulse waves with cuffs to ensure minimal effect of cuff pressure on hemodynamics. Blood pressure was measured using the cuffs on the upper arm.

CAVI was calculated based on the stiffness parameter β theory [25,26] using the following formula: where Ps is the systolic blood pressure, Pd is the diastolic blood pressure, PWV is the pulse wave velocity, ∆P is Ps−Pd, ρ is blood density, and a and b are constants.

PWV was obtained by dividing the vascular length by the time taken for the pulse wave to propagate from the aortic valve to the ankle and measured by applying cuffs on the upper arms and ankles. All measurements and calculations were performed automatically using the VaSera CAVI instrument. The mean coefficient of variation of CAVI was 3.85 [16], and CAVI has good reproducibility [27] for clinical use because 5% is the accepted limit for clinical laboratory testing. Based on data from healthy individuals (15,966 females and 16,661 males), the standard value for CAVI was set as ≤8.9 on the measuring instrument. Therefore, we defined low CAVI as ≤8.9 and high CAVI as >8.9.

Statistical analysis

SPSS software (version 22.0; IBM, Armonk, NY) was used for statistical analysis. The sample size was calculated based on the percentages of Grade 1 ISSNHL between high-CAVI and low-CAVI patients with a two-sided alpha level of 0.01 and statistical power of 85%. The percentage of Grade 1 ISSNHL patients and that of patients experiencing no change were compared between the high-CAVI and low-CAVI groups. Correlations among the various parameters described above were examined using the simple linear regression analysis, unpaired and Welch’s t-tests for continuous variables, and the chi-squared test for categorical variables. Data were fitted to the Cox proportional hazard model with complete recovery or marked improvement as the outcome. Significant variables in the univariate analysis were used as covariates for multivariate analysis. Age and CAVI were correlated and thus considered potential confounders. In addition, age had been reported as a significant variable [2,28], and CAVI was the focus of this study. Time until hearing stabilized, complete hearing recovery or dropout was used in the Cox model.

Results are presented as mean ± standard deviation and statistical significance was set at p-values of <0.05.

## Results

**Figure 1:**
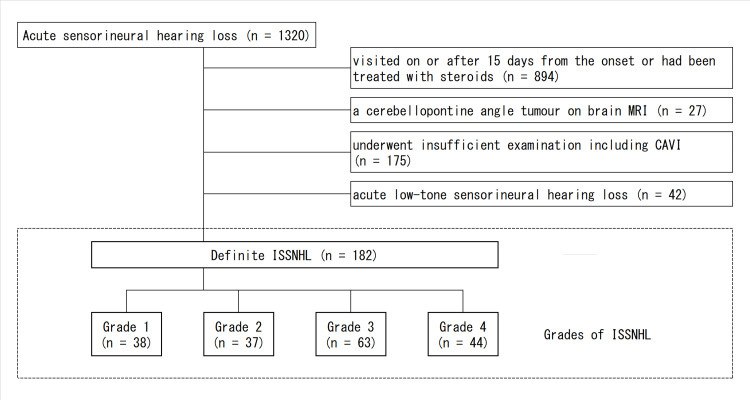
Patient eligibility and study flow MRI, magnetic resonance imaging; CAVI, cardio-ankle vascular stiffness index; ISSNHL, idiopathic sudden sensorineural hearing loss

Of the 1320 patients with ISSNHL who visited our clinic, we excluded those who visited on or after day 15 from disease onset or had been treated with steroids at a previous clinic (n=894); those who were diagnosed with cerebellopontine angle tumor by brain magnetic resonance imaging (n=27); those who underwent insufficient examination, including CAVI assessment (n=175); and those who had acute low-tone sensorineural hearing loss (n=42) (Figure 1).

Of the 175 patients who underwent insufficient examination, including CAVI assessment, 25 did not have CAVI or had an inconclusive CAVI value, and the remaining 150 patients either faced challenges in adhering to the scheduled pace of hearing tests or discontinued their visits before achieving complete recovery or stabilization of their hearing.

The sample size was calculated based on the percentages of Grade 1 ISSNHL between high-CAVI and low-CAVI patients. The calculation yielded a sample size of 72 patients in each group with a two-sided alpha level of 0.01 and a statistical power of 85.0%. Finally, 182 patients with definite ISSNHL (72 high-CAVI and 110 low-CAVI patients; mean age, 59.3±15.3 years; 86 women and 96 men) were enrolled, as patients with high CAVI and those with low CAVI visited randomly. All the patients were administered a short course of corticosteroids (prednisolone 1 mg/kg per day, with a maximum dose of 60 mg per day) for 10 days. No bilateral ISSNHL was observed during the study period.

Characteristics and test results of the low-CAVI and high-CAVI groups

In the 182 patients included, CAVI was assessed 8.3±9.9 days after initial PTA. Patients in the high-CAVI group were significantly older than those in the low-CAVI group at diagnosis (Table 3).

**Table 3:** Characteristics and test results of the low-CAVI and high-CAVI groups BMI: body mass index; CAVI: cardio-ankle vascular index; PTA: pure-tone average (arithmetic mean of the five frequencies of 250, 500, 1000, 2000, and 4000 Hz); dB: decibel; bpm: beats per minute †Unpaired t-test, ‡chi-squared test, ††Welch’s t-test *: p <0.05, **: p < 0.01

	low CAVI group (n = 110)	high CAVI group (n = 72)	p
Age (years)	52·2 ± 14·8	70·3 ± 7·5	< 0·001†**
Sex			
Female	59 (53·6%)	27 (37·5%)	0·033‡*
Male	51 (46·4%)	45 (62·5%)	
Height (cm)	162·6 ± 9·4	161·3 ± 7·9	0·33†
Weight (kg)	63·5 ± 13·2	61·8 ± 11·0	0·37†
BMI (kg/m^2^)	23·9 ± 3·9	23·7 ± 3·5	0·7†
Blood pressure (mmHg)			
Systolic	124·3 ± 15·4	138·5 ± 20·8	< 0·001††*
Diastolic	74·8 ± 10·8	79·4 ± 10·4	0·005†
Heart rate (bpm)	68·2 ± 10·3	67·2 ± 9·8	0·5†
CAVI	7·65 ± 0·99	9·98 ± 0·83	< 0·001†**
Medical histories			
Diabetes mellitus	13 (11·8%)	22 (30·6%)	0·0017‡**
Hypertension	26 (23·6%)	31 (43·1%)	0·0057‡**
Dyslipidaemia	13 (11·8%)	19 (26·4%)	0·012‡*
Initial PTA (dB)			
Unaffected side	18·3 ± 10·2	27·5 ± 11·1	0·005†**
Affected side	62·3 ± 28·0	73·7 ± 24·1	< 0·001†**
Final PTA (dB)			
Unaffected side	17·2 ± 10·5	27·6 ± 11·6	< 0·001†**
Affected side	41·3 ± 28·2	48·5 ± 25·5	0·081†
Vertigo	40 (36·4%)	16 (22·2%)	0·04‡*
Time until hearing stabilized or complete recovery (days)	135·5 ± 194·5	87·5 ± 116·0	0·039††*

**Figure 2:**
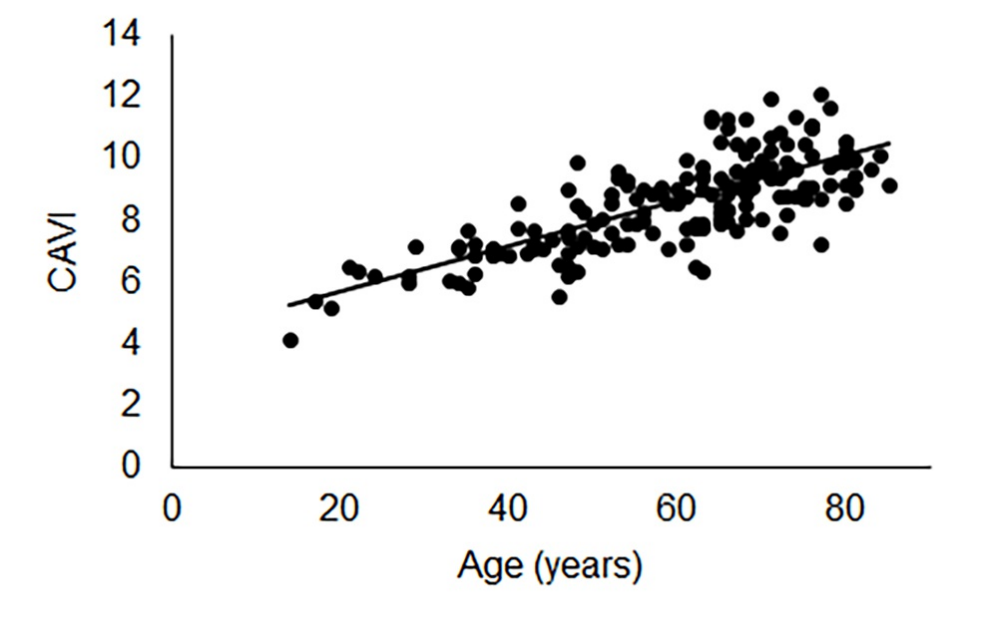
Correlation between age and CAVI on the unaffected side A positive linear correlation was noted between age and CAVI on the unaffected side. CAVI: cardio-ankle vascular stiffness index

**Figure 3:**
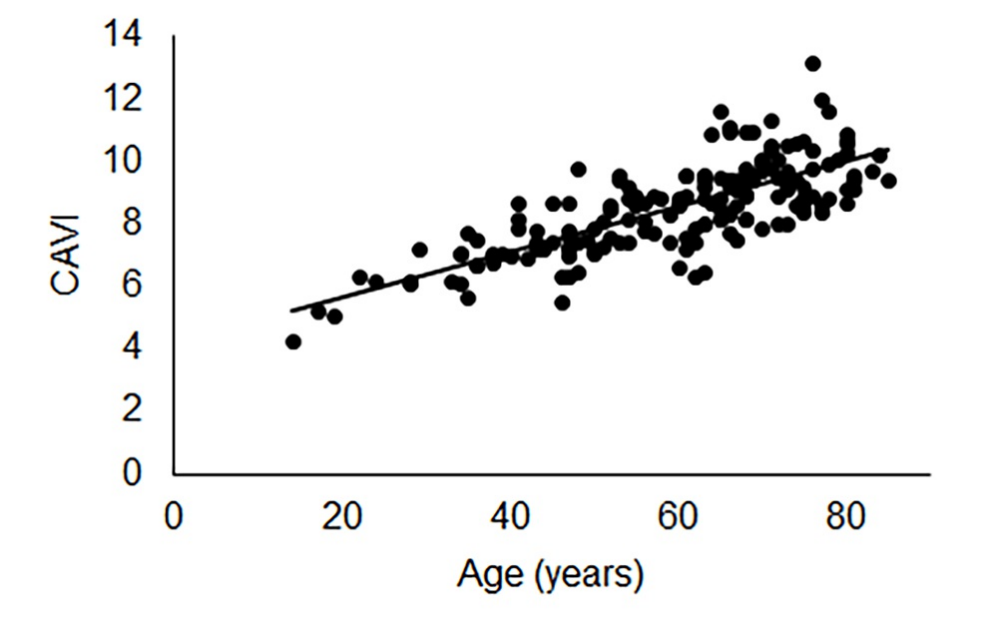
Correlation between age and CAVI on the affected side A positive linear correlation was noted between age and CAVI on the affected side. CAVI: cardio-ankle vascular index

There was a positive linear correlation between age and CAVI on both unaffected (y=0.074+4.22, R2=0.59, p<0.001; Figure 2) and affected (y=0.073+4.21, R2=0.58, p<0.001; Figure 3) sides.

The percentage of patients with vertigo or a medical history of diabetes, hypertension, or dyslipidemia was lower in the high-CAVI group than in the low-CAVI group. Pure-tone audiometry was performed during the initial examination. The initial PTA was higher in the high-CAVI group than in the low-CAVI group on both the affected and unaffected sides. The final PTA on the unaffected side was higher in the high-CAVI group than in the low-CAVI group, whereas those on the affected sides were comparable (Table 3). The time to hearing stabilization or complete recovery was longer in the low-CAVI group than in the high-CAVI group.

**Figure 4:**
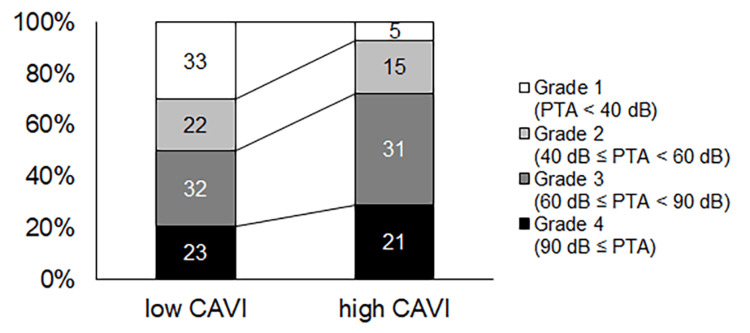
Grades of ISSNHL The percentage of Grade 1 hearing loss in PTAs of ISSNHL was smaller in the high-CAVI group than in the low-CAVI group (p<0.001). ISSNHL: idiopathic sudden sensorineural hearing loss; PTA: pure-tone average; CAVI: cardio-ankle vascular stiffness index

The percentage of Grade 1 ISSNHL was smaller in the high-CAVI group than in the low-CAVI group (p<0.001) (Figure 4).

**Figure 5:**
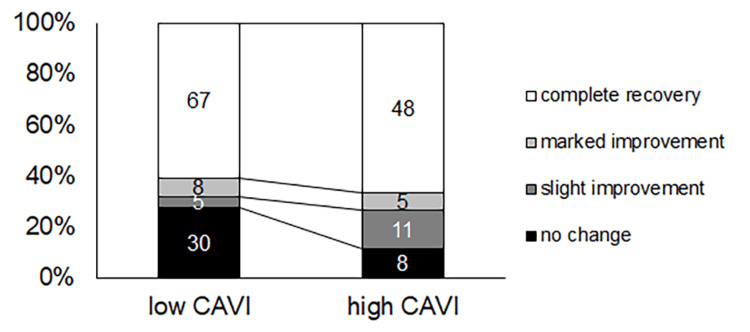
The level of hearing recovery The percentage of patients experiencing no change was smaller in the high-CAVI group than in the low-CAVI group (p=0.0087). ISSNHL: idiopathic sudden sensorineural hearing loss; PTA: pure-tone average; CAVI: cardio-ankle vascular stiffness index

In contrast, the percentage of patients experiencing no change was smaller in the high-CAVI group than in the low-CAVI group (p=0.0087) (Figure 5).

Univariate and multivariate analyses of factors affecting outcomes

Patients whose condition completely recovered or markedly improved were of comparable age to those whose condition slightly improved or experienced no change. Among patients showing complete recovery or marked improvement in hearing, the percentage of patients with Grade 4 ISSNHL and the presence of vertigo was lower, the time to hearing stabilization or time to complete recovery was shorter, and the percentage of patients with hypertension was higher than that among their counterparts (Table 4).

**Table 4:** Univariate analyses of factors between patients with complete recovery or marked improvement in hearing and patients with slight or no improvement CAVI: cardio-ankle vascular index; ISSNHL: idiopathic sudden sensorineural hearing loss †Unpaired t-test, ‡chi-squared test, ††Welch’s t-test *: p <0.05, **: p < 0.01

	Complete recovery or marked improvement (n = 128)	Slight or no improvement (n = 54)	p
Age (years)	59·5 ± 15·2	59·1 ± 15·7	0·85†
Sex			
Female	62 (48·4%)	24 (44·4%)	0·62‡
Male	66 (51·6%)	30 (55·6%)	
Grade of ISSNHL			
Grade 1-3	105 (82·0%)	33 (61·1%)	0·0026‡**
Grade 4	23 (18·0%)	21 (38·9%)	
Grade of ISSNHL			
Grade 1, 2	58 (45·3%)	17 (31·5%)	0·083‡
Grade 3, 4	70 (54·7%)	37 (68·5%)	
CAVI			
Low	75 (58·6%)	35 (64·8%)	0·43‡
High	53 (41·4%)	19 (35·2%)	
Vertigo			
Absent	97 (75·8%)	29 (53·7%)	0·0032‡**
Present	31 (24·2%)	25 (46·3%)	
Diabetes mellitus			
Absent	105 (82·0%)	42 (77·8%)	0·51‡
Present	23 (18·0%)	12 (22·2%)	
Hypertension			
Absent	82 (64·1%)	43 (79·6%)	0·039‡*
Present	46 (35·9%)	11 (20·4%)	
Dyslipidaemia			
Absent	103 (80·5%)	47 (87·0%)	0·21‡
Present	25 (19·5%)	7 (13·0%)	
Time until hearing stabilized or complete recovery (days)	84·1 ± 131·3	193·3 ± 218·7	0·0011††**

Given the number of patients in both groups (n=54), we included five covariates in multivariate analysis. ISSNHL grade (Grade 4 or others), the presence or absence of vertigo, and hypertension were set as covariates because they were significant in the univariate analysis. Age and CAVI were also set as covariates, although they were not significant. In the Cox proportional hazards model, Grade 1-3 ISSNHL (hazard ratio {HR}=0.49), younger age (HR=0.98 for every 1-year increase), high CAVI (HR=1.81), and hypertension (HR=1.92) contributed to complete recovery or marked improvement in hearing (Table 5).

**Table 5:** Results of the multivariate analysis fitted to the Cox proportional hazards model that contribute to complete recovery or marked improvement in hearing ISSNHL: idiopathic sudden sensorineural hearing loss; dB: decibel; HR: hazard ratio; CI: confidence interval; ref: reference; CAVI: cardio-ankle vascular index *: p <0.05, **: p < 0.01

	HR	95% CI	p
Age at diagnosis			
Increases 1 year	0·98	0·97 - 0·99	0·011*
Grade of ISSNHL			
Grade 1-3 (ref)			
Grade 4	0·49	0·31 - 0·79	0·0032**
CAVI			
Low (ref)			
High	1·81	1·15 - 2·84	0·0097**
Vertigo			
Absent (ref)			
Present	0·76	0·50 - 1·17	0·21
Hypertension			
Absent (ref)			
Present	1·91	1·29 - 2·85	0·0014**

## Discussion

This study revealed the factors predictive of better hearing improvement in ISSNHL: high CAVI, lower age, and less severe ISSNHL. In addition, ISSNHL in patients with high CAVI could be more severe but may have a better prognosis than that in their counterparts with different characteristics. This report is the first to describe a disease in which patients with high CAVI (i.e., stiffer and less elastic arteries) have a better prognosis than their counterparts.

Previous reports that did not include CAVI indicated that more severe ISSNHL had a poorer prognosis [28,29]. In addition, more advanced or more severe lesions have been observed in patients with cardiovascular [20,21], cerebrovascular [18,19], and macular disorders [30] with high CAVI than in their counterparts, because diseases in patients with stiff and low-elastic arteries are difficult to treat. However, in our study, the prognosis of patients with high CAVI was better than that of patients with low CAVI, despite older age and more severe ISSNHL. To explain this discrepancy, we hypothesized that different etiologies are involved in patients with low and high CAVI, as similar prognoses are expected in ISSNHL cases of the same etiology and severity. This hypothesis is supported by the following points.

First, patients with high CAVI were older than those with low CAVI at diagnosis. This finding was expected as CAVI values increase with age. However, ISSNHL is more difficult to treat in older than in younger patients [2,28]. The better prognosis in older patients with high CAVI might be due to ISSNHL mainly caused by vascular disorders, and a worse prognosis in younger patients with low CAVI might be due to ISSNHL of mainly viral etiology.

Second, the time to hearing stabilization or complete recovery was longer in the low-CAVI group than in the high-CAVI group, suggesting that ISSNHL in the high-CAVI group was relatively transient. This result is contradictory, as more severe ISSNHL usually requires a longer period for hearing improvement or recovery [28,29]. However, the different disease etiologies in the high-CAVI and low-CAVI groups may account for this finding.

Based on these findings, the percentage of ISSNHL due to vascular disorders is likely higher in patients with high CAVI than in those with low CAVI, allowing for greater hearing improvement in the high-CAVI group than in the low-CAVI group.

The prognosis for hearing recovery is affected by age, vertigo at onset, hearing loss severity, audiometric configuration, and the time between hearing loss onset and treatment [2,28]. Herein, PTA in patients with high CAVI improved more significantly than in those with low CAVI, although these patients were older. In the Cox proportional hazards model, CAVI, ISSNHL severity, age, and hypertension were associated with hearing recovery in ISSNHL. This finding is consistent with that of previous reports, showing that severe ISSNHL has a poor prognosis [2,28,29]. In the univariate analysis, there were more patients with accompanying vertigo in the complete recovery or marked improvement groups. However, vertigo was not associated with outcomes in the multivariate analysis, suggesting that it is less relevant in this context than other significant covariates. Patients with hypertension had a relatively good hearing prognosis; this finding supports the hypothesis that patients with a better prognosis had a higher percentage of ISSNHL due to vascular disorders. The reason why age and CAVI were not significant in the univariate analysis is that these factors were correlated; therefore, they were considered confounders of each other. However, the prognosis of ISSNHL worsened with increasing age, and, conversely, it improved with increasing CAVI. Age and CAVI were significant covariates because confounding was resolved in the multivariate analysis.

This study has some limitations. First, the present findings preclude drawing definitive conclusions about ISSNHL in patients with high CAVI caused by vascular disorders resulting in good prognosis, in contrast to those with low CAVI caused by viral infections resulting in poor prognosis. Second, CAVI is an index of vascular stiffness and elasticity and cannot directly determine any disease etiology.

## Conclusions

ISSNHL in patients with high CAVI was more severe but had a better prognosis than that in those with low CAVI. High CAVI, hypertension, younger age, and initial PTA <90 dB were associated with a good prognosis. CAVI can be considered an additional indicator for assessing the prognosis of ISSNHL.
